# Indian fathers are involved in nurturing healthy behaviours in adolescents: A qualitative inquiry

**DOI:** 10.1186/s12889-024-17634-7

**Published:** 2024-01-04

**Authors:** Neha Rathi, Sangeeta Kansal, Anthony Worsley

**Affiliations:** 1 Department of Home Science, Mahila Maha Vidyalaya, Banaras Hindu University, 221005 Varanasi, Uttar Pradesh India; 2 Department of Community Medicine, Institute of Medical Sciences, Banaras Hindu University, 221005 Varanasi, Uttar Pradesh India; 3 School of Exercise and Nutrition Sciences, Deakin University, 3220 Geelong, VIC Australia

**Keywords:** India, Adolescents, Fathers, Diet, Physical activity, Parenting

## Abstract

**Background:**

Indian adolescents exhibit unhealthy food behaviours and inactive lifestyles which increase their risk of developing obesity and associated negative health consequences. The family food environment represents a vital setting to nurture healthy lifestyle behaviours in adolescents, with parents influencing their adolescents’ dietary and physical activity behaviours. Yet, much of the existing evidence exploring parental influences predominantly focuses on mothers while fathers’ engagement in instilling healthy dietary and physical activity behaviours is understudied, more so in the context of developing economies like India. Therefore, this qualitative study was designed to understand Indian fathers’ views on instilling healthy behaviours in their children.

**Methods:**

Convenience sampling along with snowball sampling techniques were employed to recruit fathers of adolescents aged 10–19 years from Kolkata city, India. Informed by the research aim and review of literature, an interview guide was developed and pre-tested. Interviews were carried out either in person or virtually (Zoom/telephone) in English/Hindi/Bengali as per the preference of the participants. All interactions were audio recorded, transcribed verbatim, and translated to English for the purpose of data analysis. The transcripts were analysed thematically using NVivo software program. Themes were identified using both inductive and deductive approaches.

**Results:**

A total 36 fathers participated in the interviews. Seven main themes were identified: (i) Involvement of fathers in adolescent upbringing (i.e. engagement in meal preparation, food shopping, educational activities, physical activity); (ii) Family food environment (i.e. setting food rules, having meals with children, making food available); (iii) Challenges to instilling healthy behaviours in adolescents (i.e. adolescents’ sedentary lifestyle and liking for unhealthy foods); (iv) Barriers to routine involvement in adolescent upbringing (i.e. time constraints due to paid employment, poor socio-economic status); (v) Adolescent nutrition education: (vi) Dual burden of malnutrition (i.e. awareness of malnutrition, no knowledge about government-led health programs for adolescents); (vii) Paternal knowledge.

**Conclusions:**

The emerging themes reveal that Indian fathers played a crucial role in instilling healthy dietary and physical activity behaviour in their adolescents through various parenting practices such as purchasing nutritious food, enforcing food rules, disseminating nutrition-related knowledge, and encouraging adolescents to participate in moderate-to-vigorous intensity outdoor sports. This provides strong support for the inclusion of fathers in sustainable family-focused lifestyle interventions to maximise the nurturing care required by adolescents as well as assist in normalising the representation of fathers in health and welfare policies designed for adolescents.

## Background

Adolescence is a vulnerable life phase associated with rapid growth and development and therefore, enhanced nutritional requirements [1]. The dietary intakes of Indian adolescents are considered suboptimal, with minimal fruit and vegetable intakes [2] and rampant consumption of energy-dense, nutrient-poor foods [3, 4]. In addition, adolescents mostly lead a sedentary lifestyle [5]. Globally, 81% of school-going children aged 11–17 years were found to be insufficiently physically active in 2016 while the overall prevalence of insufficient physical activity among Indian adolescents was 73.9% in 2016 [6]. Accumulating evidence suggests that unhealthy lifestyle behaviours result in adolescent obesity and associated adverse health complications [7–9]. The recently conducted national survey [10] found that nearly one-quarter of the sample (24% females; 22.9% males) aged 15–49 years were either overweight or obese (BMI ≥ 25.0 kg/m^2^). Lifestyle behaviours formed in adolescence often track into adulthood with significant implications for future health [1]. Therefore, it is important to instil healthy lifestyle behaviours at an early age.

Family diet may partly contribute to this emerging obesity epidemic with parents’ knowledge, attitudes, beliefs, food habits, and food parenting practices directly and indirectly impacting their adolescents’ food behaviours [11–13]. In the same vein, parents have shown to exert a social influence on adolescents’ physical activity levels either through parental support or behaviour modelling [14–16]. The existing body of evidence about parental influences on children’s lifestyle behaviours is primarily focussed on mothers [17–19]. This is possibly the result of conventional gender roles in which mothers are the primary caregivers and food gatekeepers [20–22] while fathers are the household breadwinners [23–25]. However, the last few decades have witnessed a notable sociocultural change in family structures, demonstrated by global increases in maternal employment in the paid workforce [26, 27] and the simultaneous emergence of nuclear families, particularly in urban India [28].

This radical change in the family dynamics in India and worldwide [29–31] has led to a rise in fathers’ engagement in child rearing activities [32–36], which may influence the dietary patterns and diet-related health outcomes of family members [37]. Indeed, the literature on parenting highlights the positive influence of fathers’ caregiving duties on their adolescents’ health behaviours [36, 38–40]. For example, favourable food parenting practices implemented by Latino fathers were associated with increased intakes of fruits and vegetables in their adolescents [38]. Similarly, Australian fathers’ reinforcement was significantly associated with physical activity in children aged 5–12 years [41]. Likewise, increased father involvement that is a positive father-child relationship was associated with a reduced risk of substance use behaviours in American adolescents [42]. Perhaps, that is why some researchers have reinforced the need for engaging fathers for effective delivery of family-focused lifestyle interventions [12, 43–47].

Although empirical evidence on the paternal influences on the dietary and physical activity behaviours of adolescents is expanding, very limited evidence exists about the influence of Indian fathers. Moreover, health-promoting behaviour change interventions designed for children and adolescents typically involve mothers whilst inadvertently ignoring the paternal influence [47–49]. Likewise, even the government welfare policies and programs for the adolescents devote very little attention to fathers as they specifically target mothers, who are commonly believed to be the main caregiver [44]. Nevertheless, emerging evidence from the developed economies suggest that father-focussed interventions have been successful in improving children’s physical activity levels and dietary outcomes [50–53]. Therefore, in order to facilitate healthy living among 253 million Indian adolescents, it is important to understand Indian fathers’ perceptions of cultivating healthy behaviour in their children. Insights gained from this research inquiry may support the engagement of fathers in sustainable family-focused lifestyle interventions as well as assist in normalising the representation of fathers in health and welfare policies designed for adolescents.

## Methods

### Study design

The present study adopted a qualitative research study design [54] which was informed by the social constructivism framework [55]. This framework considers how experiences are socially and culturally constructed and allows researchers to draw novel insights on the ways in which individuals communicate with other members of society [55]. Using this social lens, fathers’ experiences of adolescent upbringing was explored in this inquiry. Fathers’ experiences are mostly determined by the society in which they live and are created through their interactions with other members of that society [56]. The research protocol for this inquiry was approved by the Institutional Ethical Committee at Institute of Medical Sciences, Banaras Hindu University (Dean/2021/EC/3006). The research protocol for this inquiry has been described as per the Consolidated Criteria for Reporting Qualitative Research (COREQ)– a 32 item checklist [57].

Within the context of the current inquiry, NR is an early-career female researcher with expertise in public health nutrition and qualitative research techniques. SK is a female medical practitioner with a rich experience in family medicine and adolescent nutrition. AW is a senior male researcher with an extensive experience in consumer food and nutrition research. All three authors were involved in conceptualizing the research, NR was responsible for data collection, analysis, and writing while SK and AW reviewed the analysis and reporting of the findings. The engagement of authors from varied professional backgrounds limited the likelihood of any personal or disciplinary biases during the processes of data interpretation and manuscript writing.

### Recruitment of participants

Advertisements on different social media platforms (e.g. Facebook, Twitter, WhatsApp, Linkedln) were used to invite participants for the study. Fathers who had at least one adolescent aged between 10 and 19 years were eligible to participate in the present inquiry. Both convenience and snowball sampling techniques were employed to recruit participants from Kolkata, India. Kolkata is a cosmopolis and therefore our study participants were likely to represent urban Indian fathers. Fathers expressed their interest in participation to the lead author (NR) via email or telephone. Subsequently, a plain language statement explaining the research procedure along with a consent form was provided to all the interested participants via email or in person. Upon receiving consent from the fathers, they were asked to specify their preferred time, date, medium of interview (i.e. virtual/face-to-face) and location of interview (in case of physical interview). Qualitative researchers often endorse the use of telephone/online interviews as an equally viable option to face-to-face interviews because data quality is unaffected by the mode of data collection [58–60, 61, 62].

### Data collection

Based on the research aim and review of previous studies [37, 63, 64], an interview guide comprising a series of open-ended questions (Table 1) was designed and pre-tested with five fathers. No modifications were made in the interview guide as the five respondents were able to answer all the questions.

**Table 1:** Interview Guide

Q1	If I were to spend a normal day with you, what kind of things would I see you doing with your adolescent?
Q2	How do you support your wife in general? (*Do you cook for your adolescent? Do you engage in grocery shopping/buying food for the adolescent?*)
Q3	How would you feel if your wife asked you to help her by cooking for the adolescent?
Q4	What are the challenges you face while taking care of your adolescent (e.g. feeding the adolescent)?
Q5	Does anything inhibit you from being involved in your adolescent’s life?
Q6	What would be your response if you were asked to be involved in daily childcare activities?
Q7	In your opinion, what can be done to improve adolescent dietary practices?
Q8	Are you aware of child malnutrition (both underweight & obesity)? Have you ever attended any program or meeting on malnutrition? Did you come to know about malnutrition on social media?
Q9	What sort of information or ideas would you be interested in knowing more about with respect to ideas on how to raise healthy, active adolescents?

NR conducted all the interviews either over Zoom/telephone or face-to-face as per the preference of the fathers. In-person interviews were carried out in the interviewee’s workplace. All the interviews were conducted between December 2021 and December 2022 by NR, who had no prior relationships with any of the interviewees. At the onset of the interviews, all the participants were notified that the interviews would be audio recorded and that all the shared information would be treated confidentially. They were further informed that their participation was voluntary, and they were free to leave the study at any time before the data was analysed thematically. According to the choice of the interviewee, the interviews were either conducted in the local language (Bengali or Hindi) or English (NR is well versed in all three languages) until thematic saturation was reached [65]. A total of 36 interviews were conducted: out of these 10 interviews were completed virtually (5 over telephone; 5 over Zoom) and the remaining 26 interviews were carried out face-to-face. The average duration of the interviews was 34 min ranging from 21 to 45 min. Upon completion of the interviews, all participants received a coffee mug as a compensation of their time.

### Data analysis

Thematic analysis was carried out concomitantly with the interviews to maintain a balance between the two research processes [66]. A professional transcription service was utilised to transcribe verbatim and translate the audio recordings to English. A subset of five transcripts was verified by the interviewer (NR) for accuracy. Following transcription, all interviewees were invited to review the transcripts. None of them provided any feedback. Subsequently, the transcripts were transferred to the NVivo (Version 12) qualitative analysis software program (QSR International Pvt Ltd. 2018) for the purpose of data analysis.

Theme generation was informed by the Template Analysis Technique which comprises both inductive (codes and themes are identified directly from the raw data) and deductive coding (codes and themes are identified based on review of literature and research aim) approaches [67]. An initial sample of ten randomly selected transcripts was analysed for the development of the *‘a priori’* template and this coding template was compared and discussed among the two coders (SK and NR). Any disagreement between the two coders was resolved through discussion and consultation with the third author (AW). Following the initial development of the template, NR reviewed all the remaining transcripts wherein the *‘a priori’* template was modified (i.e. some codes were merged, removed or renamed for clarity). Throughout the coding process, multiple meetings between the authors were held to discuss template development process at length and the generation of conceptual themes. Three professionals (i.e. one nutritionist, one social psychologist, and one special educator) were hired to independently analyse three transcripts each to confirm inter-rater reliability [68]. The conceptual themes and the associated narratives from the participants’ accounts have been provided in the Results section.

## Results

### Socio-demographic characteristics of the sample

Thirty-six fathers participated in the interviews whose mean age was 45.5 years (ranging from 32 years to 53 years) and all of them were employed. Three quarters of the sample (27/36) were university educated while 23 fathers belonged to the upper social class. Two-thirds of the fathers (24/36) came from single-earning families and nearly half of the sample (19/36) represented a nuclear family. The socio-demographic characteristics of the fathers and their families are presented in Table 2.

**Table 2:** Socio-Demographic characteristics of fathers and their families (*N* = 36)

Socio-demographic items		N
Age	31–40 years	6
	41–50 years	21
	51–60 years	9
Education	Primary School	1
	Middle School	4
	High School	4
	Bachelors	17
	Masters	9
	Doctorate	1
SES*	Upper	23
	Upper Middle	2
	Middle	6
	Lower Middle	5
	Lower	0
Family structure**	Joint	17
	Nuclear	19
Family employment structure	Single earning	24
	Dual earning	11
	Triple earning	1

*SES: socio-economic status was calculated according to the B.G. Prasad Scale [69]

** Nuclear family: Two generations (i.e. parents and their children) staying together under one roof; Joint family: A large undivided family where three generations or more live together in a common house [70]

### Themes and sub-themes

Seven major themes were identified through thematic analyses of the 36 interviews associated with the role of fathers in instilling healthy behaviours in their adolescents: (i) Involvement of fathers in adolescent upbringing; (ii) Family food environment; (iii) Challenges to instilling healthy behaviours in adolescents; (iv) Barriers to routine involvement in adolescent upbringing; (v) Adolescent nutrition education: (vi) Dual burden of malnutrition; (vii) Paternal knowledge These themes and the sub-themes are discussed below, and illustrative quotes are presented in Table 3.

**Table 3:** Themes and sub-themes associated with the role of fathers in instilling healthy behaviors in their adolescents

Themes	Sub-themes	Illustrative quotes
***Theme 1 - Involvement of fathers in adolescent upbringing***	Meal Preparation	*“Inside the house kitchen work, my involvement is nearly zero. But in other things, I make them* (children) *make their bed and all and not to tire my wife out. Going and getting groceries, I am 100% involved. Shopping for the kids also I am fully involved.” (*FA2) *“I don’t get into the kitchen primarily. My role is outside the kitchen.”*(FA28) *“Frankly speaking, I don’t do anything, whatever cooking, feeding. I do one thing; I make sure that I give them 2 bottles of water. I fill water bottles and keep them on their table. Two bottles for sure, for both. This is the only thing that I do and nothing as such. I give them fruits sometimes otherwise I don’t indulge much in getting them fed…. My wife mostly indulges in cooking and feeding. In teaching, yes, sometimes in morning, I do sit with her. In evening, I ask her about what she studied, I generally ask her what she did, you know…”* (FA6)
	Food shopping	*“Grocery, I must take care, because my wife doesn’t go outside. So, for grocery shopping and going to the market I go and get. Or if I must get anything for my son, I will only get it.”* (FA1) *“Yeah, my role is smaller because my role is to get the stuff, to get the ingredients from the market bread, then all those spices, condiments, cheese and all that so I get from the market. Maybe a little bit of vegetable cutting…. but the cooking part is being taken care of by my wife….”* (FA9) *“I take care of grocery and the vegetables and fruit, I take care. No, I haven’t ever cooked for my son. No, I have cooked Maggi when my wife fell sick, I am not a good cook but can manage….”* (FA20)
	Educational activities	*“On a working day, to be frank I get very little free time to spend with my daughter, but what I spend with her is mainly related to her educational activities. Suppose she isn’t getting Mathematics or English work then I help her. In that case I am spending time with her related to educational activities rather than personal activities.”* (FA3) *“No, I don’t cook. Her mother only cooks….I do help her with mathematical problems but only when I get time. For instance, when I find her sitting beside me, doing mathematics, I try to help her out.”* (FA29) *“Many a times, I go to work late so that I can drop children at school. If I complete my work early, I go to receive the kids at their dispersal timings. I cook food for them on holidays.”* (FA4) *“I check upon if they have completed their schoolwork after returning from work.”* (FA10)
	Physical activity	*“In a month, 10–15 days I take my daughters for playing…. Though, this doesn’t happen nowadays, not on daily routine basis, may be on holidays.”* (FA6) *“My participation is more academic related. On weekends I take him to cricket coaching center and we practice together.”* (FA8) *“I push her, so she has certain physical activities, but I don’t push her beyond a limit. I tell her, ‘Why are you spending time over phone why don’t you go to a gym, ride a cycle?’”(*FA15) *“On weekends I take them to park and play with them.”* (FA10)
***Theme 2 - Family food environment***	Family meals	*“….Till now we have been maintaining this that we will have our dinner together at around 10:30 p.m.”* (FA24) *“We eat all meals together on Sundays…… On weekdays, I eat snacks with them in the evening if I come home early, and then I spend time with them till night. I also have dinner with them….”* (FA10)
	Food rules	*“I have strict rule for junk items. Maggi noodles are available only on Sunday mornings. chips etc.”* (FA2) *“….As he is growing older, he is starting to prefer outside food, junk foods so it is not like that we don’t allow him, we allow him but in restricted way. We have decided that we shall give you fast food just once a week or may be maximum twice a week but not more than that….”* (FA7)
	Availability of food	*“I’ve stopped getting cold drinks inside the house fully.”* (FA12) *“I personally avoid cold drinks and try to make them understand the problems that I have faced. So, I avoid keeping a stock of such food items in the refrigerator to maintain this……”* (FA35)
***Theme 3 - Challenges to instilling healthy behaviors in adolescents***	Disliking for healthy foods vs. liking for unhealthy foods	*“I love fruits, but she won’t eat anything other than sweet lime or cucumber so in such scenarios we have arguments at home like if every kid can have why won’t she….”* (FA23) *“Like I prefer bitter gourd and such vegetables as I have diabetes. Yes, I really do like bitter vegetables, but he isn’t very fond of them. He will have a little bit of it. He does not like all this…….”* (FA14)
	Sedentary lifestyle and exposure to screen	*“Sometimes the kids don’t listen to me. I feel like they’re stuck in the TV….”* (FA5) *“Recently the problem that we are facing is that either I must give my mobile phone to her or her mother’s mobile phone to her to play games. Otherwise they claim to have been bored. I try to entertain her by buying her carrom board because it is a very useful game because it improves concentration and hand eye coordination. Sometimes I try playing chess or scrabble with her.”* (FA33)
***Theme 4 - Barriers to routine involvement in adolescent upbringing***	Paid employment	*“See work is the only barrier since we are into family business, I need to leave for office by 10. I come back by 6:30 − 7. These eight hours are being spent in the office so when do you spend time with kids. Work barrier is there and will always be there because the nature of work is like that.”* (FA9) *“In daily routine, time spent is very less because I leave for work at 8:30 − 9:00 am and come back at 8:00 to 9:00 pm so not much time is spent with children.”* (FA6)
	Financial status	*“I come from a middle-class family. I am not able to emulate the feeding practice pattern of other wealthy and privileged families. Most of the time, I am not able to feed my children luxuriously but feed him with the necessary food items like rice, vegetables, pulses, etc.”* (FA4) *“I want to do so many things for her, but I can’t afford to do so.”* (FA29) *“How will I be satisfied? Well of course money is really a big factor. I want to teach her computer etc etc, but money is a big factor”* (FA23) *See we have so many things that we want to do but one of the most important barriers is the monetary barrier like even if I want to do something for her, I won’t be able to do that.* (FA26) *“For me time and money are an issue. I feel bad when they ask for something and I can’t buy it, like fruits, books….”* (FA5)
***Theme 5 - Adolescent nutrition education***		*“Another thing is that she was never fond of eating vegetables especially. But I made her understand that she must eat vegetables for the sake of her growth.”* (FA3) *“….I just explain to them which food is good, which they should avoid and eat. They avoid all beverages; I talk to them about what to eat and what to avoid only and what will keep them healthy…”* (FA1)
***Theme 6 - Dual burden of malnutrition***	Awareness of undernutrition and obesity	*“Obesity, I’ve heard a lot. Because in my younger days, I was with someone who was obese. So, from that time, it was a trigger point, and I knew I never wanted to see me or my family like that. That was a trigger point that I decided to make a plan and not give junk food and if I give it will be limited….”* (FA32) *“Basically, what is obesity, the reasons for it, the food habit, lifestyles and that’s why people are obese. I have seen on social media, randomly it comes in between, like viral links….”* (FA2) *“I have heard about many cases regarding it* (undernutrition) *while I travel by train.”* (FA25) *“Yes, I have heard about malnutrition of children. I have seen kids who are suffering from underweight and obesity. I have often heard this from doctors. Also, I did come to know about this from television and conversation of social gatherings in my locality.”* (FA4)
	No awareness regarding government programs	*“No, I haven’t attended any meeting on undernutrition.”* (FA5) *“No, I did not attend such meetings or programs on malnutrition. I really have no clue about this.”* (FA4) *“No, I did not attend any government program on malnutrition. Never heard of it.”* (FA35)
***Theme 7 - Paternal knowledge***		*“I would ask a doctor, what are the things that, I as a parent, am liable to perform to make my children mentally and physically fit. I would also ask the ways to improve my child’s memory power. Does she have to follow a diet chart? How much physical activity is required for her on a daily basis?”* (FA36) “*Regarding her food habit one thing that I would like to know is what food should we feed her when she is having stomach upset or vomiting or she’s having fever. Which are the food items that will help her recover fast??”* (FA26)

#### Theme 1 - Involvement of fathers in adolescent upbringing

##### Meal preparation

The majority of fathers (29/36) did not engage in meal preparation for their adolescents on a regular basis mainly because they did not know how to cook. They regarded cooking as the primary responsibility of the mother.

*“If you are talking about helping in cooking, I am sorry because I don’t know cooking. I can prepare tea, Horlicks* (Brand name for a health drink), *warm milk, this much also I did not know before. After Harsh’s* (Name of the adolescent ) *birth, I have learnt these things. Before him, I did not even know how to light the gas stove….”* (FA7).

Nevertheless, there were few fathers (7/36) who cooked mainly on weekends as time was available or when the mother was ill. While cooking on holidays, fathers involved their adolescents too to make it a recreational activity as well as to instil essential living skills in them.

*“See I am not much into cooking; I can prepare Maggi* (Brand name for instant noodles) *of course. So, when her mother is not at home, we* (father and the daughter) *prepare Maggi together. She cuts the vegetables, that’s enjoyable, fun rather you can say. Once I prepared chicken au gratin for her, I saw from YouTube and made, that’s all. It is basically participating in fun activity and tried to cultivate interest in cooking because I know she will have to move out and she won’t have problems.”* (FA15).

##### Food shopping

Although fathers did not play a major role in food preparation for their children, but nearly all of them (34/36) were actively involved in purchasing groceries and other food stuff for the household and the family members as demonstrated by the following two quotes:

*“She* (wife) *doesn’t have to pay any attention to getting in groceries and all that. That is my department totally. Whatever is required in house, that I look after…”* (FA18).

*“See I’m not much into cooking for the kids yeah maybe once in a blue moon I make something for them let’s say a Maggi or biryani. My kids love to eat biryani, so I watch YouTube videos and make something for them but that’s it once in a month or once in 15 day types. It’s mostly my wife who makes food for the kids and yeah, my job is restricted to buying groceries buying food, fruits, vegetables, and all. I get it daily so that’s primarily my job. WhatsApp is the best medium. Whatever she wants she just puts it on WhatsApp and my job is to get that stuff from anywhere be it online, burra bazar* (Central Business District of Kolkata) *or Big Bazaar* (A supermarket chain) *or whatever it is… Basically I’m the purchase or resource manager for my house. We have this family group. Whoever needs whatever be it medicine or whatever food stuff, they just put it on the family group, and I need to arrange it.”* (FA12).

##### Educational activities

In addition to their active participation in food shopping, most of the fathers (30/36) discussed their contributions to their adolescents’ educational activities which included assisting in projects, problem solving, and dropping off at school.

*“If I come back home early that is 6–6:30 types then we spend time together…maybe if some school project is there so I help him with his school projects making the PPT, taking the printouts, getting information from the net, searching the Google and making all those life science project….”* (FA17).

##### Physical activity

The interviewees noted that they constantly encouraged their adolescents to play outdoors and engage in moderate-to-vigorous intensity sports. Nevertheless, their direct involvement in adolescents’ physical activity e.g. practicing cricket with the adolescent was mostly restricted to weekends and holidays.

*“On a Sunday, I take them to the park maybe for a horse ride or go for swimming…. On weekdays, I insist them to go downstairs and play and ride their cycles. We make sure that that they play and sweat it out every day. So, we ask them to do those skipping like things at least for 30 minutes every day then ride a cycle so that they sweat enough on a daily basis.”* (FA13).

#### Theme 2 - Family food environment

##### Family meals

Most of the fathers (31/36) lamented that consuming all meals with the adolescents was not feasible on weekdays because of the different daily routines of the family members. Despite different work schedules, the respondents claimed to consume dinner with their adolescents on a daily basis. Weekends and holidays allowed for consuming all the meals and snacks with the adolescents as narrated by the majority of the respondents.

*“On weekdays, only dinner we have together, nothing else. My routine is different, their routine is different. So, it’s only dinner. On weekends, we have all meals together.”* (FA31).

*“On holidays we have lunch together otherwise it is not possible……” (FA8)*.

##### Food rules

The study respondents discussed their imposition of food rules on adolescents. One of the common food rules identified by our fathers was prohibiting children from watching television or mobile phone while eating. Another commonly enforced food rule was related to junk food wherein junk food preparation in the household or its consumption was restricted to just one day a week. Despite imposing these rules, adolescents often flouted them as reported by some fathers.

*“I had set a rule of not allowing him to watch television while having food but have been unsuccessful because the children do not get much time for relaxation the entire day. While having lunch he does not watch television as he is at school but at night after he completes his homework, he watches television while having dinner.”* (FA35).

*“Nowadays children are attracted towards fast food like pizza, but I try to avoid it as much as possible. I have given instructions to my wife to make the food items at home once a week so that the children do not develop a habit of eating outside food. He may not follow this later but at present he does. Also, the cost of buying food outside is almost double or triple when compared to making the same item at home.”* (FA24).

Besides imposing food rules, few fathers stipulated meal timings for their teenagers as highlighted in the quote below:

*“I have fixed time for meals. For lunch, the time is fixed to 1:30 − 2:00 pm, dinner time is 9:30 − 10:00 pm. If she is at home, she must follow these timings……”* (FA36).

##### Availability of food

The fathers also talked about the availability of food items in their household. They mentioned that they refrained from keeping unhealthy foods and drinks in their kitchen or refrigerator to curtail their consumption among the teenagers.

*“I don’t keep cold drinks at home. Sweets, we generally don’t keep at home. Nowadays, biscuits and chips aren’t also kept much at home….See if you don’t make them available how will they consume it??”* (FA36).

#### Theme 3 - Challenges to instilling healthy behaviours in adolescents

##### Disliking for healthy foods vs. liking for unhealthy foods

Most of the fathers (27/36) reported that it was quite challenging for them to foster healthy eating habits in their adolescents since they did not enjoy eating green vegetables and fruits; rather, they relished the taste of high-energy, nutrient-poor foods.

*“He loves having fast foods……He doesn’t like vegetables much.”* (FA12)

*“Sometimes during dinner time, she says that she won’t eat, and she doesn’t take any food. When I asked her why she won’t eat dinner, I found out that she is eating fast food in the evening, so she won’t eat dinner. So, I try to make her understand that she shouldn’t eat fast food during the evening and have dinner and that she shouldn’t skip her dinner. She should at least have one chapati* (Indian flat bread). *So that is one challenge I face”* (FA15).

##### Sedentary lifestyle and exposure to screens

The interviewees often complained about adolescents’ excessive use of electronic devices including mobile phones, television, and tablets for their entertainment. They further noted that adolescents were mostly occupied in studying or using digital devices and therefore did not engage in indoor or outdoor sport which resulted in weight gain in some of the adolescents.

*“For the last two-three years his physical activity has gone down considerably I would say. Earlier he used to play, he used to go to club for cricket coaching and all that, which has stopped because of his studies and COVID and all that. So, this is where if he can involve himself more into physical exercises it would be better.”* (FA18).

*“My son is a little overweight. He is 13 years now and his weight is almost 50–52 kgs….I think the main reason is that he is not getting much physical activity. Going to school, coming from school, studying in front of the tutor, and then watching TV or sitting with his mobile.”* (FA1).

#### Theme 4 - Barriers to routine involvement in adolescent upbringing

##### Paid employment

Unanimously, all the fathers reported feeling guilty as they were unable to give sufficient time to their adolescents because of their long work hours. This feeling of guilt is expressed in the following quote:

*“I feel that just admitting her to a good, reputed school is not enough, good parenting and guidance is equally important. That is the place where I am lagging. This is because I cannot spend enough time with my daughter as much as required for her grooming. Spending time with children is very important. The time that we need to groom our children is not provided by our work environment. I’m seeing this because the frustration regarding work affects my relationship with my child maybe after I come back home she comes to me to ask something but I get annoyed and start scolding her….”* (FA33).

##### Financial status

Besides long office hours, fathers predominantly from middle and lower socio-economic strata, also complained about their poor financial status which hindered them from taking good care of their adolescents that is they were unable to provide healthy food like fruits as narrated below:

*“Sometimes I will get fruits. She loves fruits, I mean my daughter…Nope it’s not possible to get it daily to be honest I can’t afford that I don’t have that much money.Maybe one day I buy 5–6 oranges then there is a gap of 2 days then again, I will get fruits for home and again a gap of 2 days….like where will I get money otherwise.”* (FA10).

#### Theme 5– Adolescent nutrition education

Nearly all the fathers (32/36) provided nutrition education to their adolescents regarding the harmful effects of excessive consumption of energy-dense, nutrient-poor foods and sugar-sweetened beverages as well as the benefits associated with consumption of green leafy vegetables and fruits. The following quote exemplifies this:

*“Whenever he wants to order food or wants to eat out and things like that then I explain to him that it is not advisable to eat junk food every day because it is not good for his health or anyone’s health for that matter. We try to minimize such practices of his and improve it.”* (FA7).

#### Theme 6– Dual burden of malnutrition

##### Awareness of undernutrition and obesity

All the participants were aware of both forms of malnutrition and their consequences for adolescent and adult health. They attributed this awareness to various sources of information which included television, newspaper, social media, doctors, and personal experience.

*“I have read some articles. I’ve gone through some statistics like people in developed countries are facing adolescent obesity and all related stuff heart problems, blood pressure and juvenile diabetes and all….therefore we make sure that kids have a balanced diet so that this situation doesn’t arise.”* (FA9).

##### No awareness of government programs

None of the study participants knew of any government health program or policies to tackle obesity and undernutrition. One of the fathers was baffled with the question about government programs and mentioned that he had neither heard anything like this, nor had he received any service from the Government. Here is his quote:

*“Is there any government program on malnutrition??? No, never. No, I have never heard of it in fact. Neither has anyone reached out to me nor have I reached out to them.”* (FA7).

#### Theme 7– Paternal knowledge

Most of the fathers (31/36) were concerned about the emerging unhealthy eating habits and sedentary lifestyle in their children. They expressed keen interest in enhancing their knowledge about how to rectify their children’s unhealthy behaviours so that they can perform well at school and remain healthy throughout their lives.

*“I would love to understand more about nutrition, so that if there is scope for improvement in the daily diet, then I would try to change it or give a better lifestyle to my kids. I am open to it. Nowadays the food habits that children have is worrisome, so I would like to understand what they should eat to be active and healthy.”* (FA11).

## Discussion

This qualitative inquiry contributes to the limited work examining fathers’ beliefs and perceived roles in nurturing healthy behaviours in their adolescents. The key findings were that fathers played a vital role in adolescent upbringing through food provisioning, offering a healthy home food environment, providing nutrition education, and encouraging adolescents to lead an active life. However, fathers faced challenges while ensuring healthy upbringing of their adolescents which included adolescents’ liking for unhealthy foods and their excessive exposure to screens. Fathers lamented that full-time employment duties and poor financial status inhibited them from taking adequate care of their teenagers.

The interviewees did not play a significant role in cooking meals for adolescents as they believed they were incompetent to cook because of their lack of culinary skills. Likewise, American fathers also reported that it was not their duty to prepare or provide healthy meals to their teenagers [37]. This incompetency could be partly attributed to the fact that customarily domestic cooking and Home Economics was not offered to boys in school [71–75]. Nevertheless, our interviewees mentioned that they were responsible for purchasing groceries and fresh produce for the household. In the same vein, 24% of the multi-ethnic fathers (*n* = 51; had children aged 8–12 years) living in the US indicated that they had the primary grocery shopping responsibility for their family, while another 10% reported sharing the role with their spouses [76]. Comparable views regarding food shopping were also cited by fathers of young children in rural Pakistan [77] and Uganda [78].

Besides food shopping, fathers noted that they persistently promoted their teenagers to participate in moderate-vigorous intensity outdoor sports and also played with them over the weekends. Similarly, 19 Mexican-American fathers from four focus groups mentioned spending time with their 10- to 14-year-old adolescents in various physical activities like playing soccer and walking around the park [79]. Consistent with these findings, mothers in a UK based qualitative study acknowledged fathers as the family’s “physical activity leaders” who were largely responsible for engaging the children aged 5–6 years in various sports such as swimming, running, playing football, and cycling [80]. In similar lines, data from the US-based longitudinal study indicated that fathers’ weekly vigorous physical activity frequency was positively and consistently associated with adolescents’ (aged 10–18 years) participation in vigorous physical activities [81]. All these findings suggest that paternal-child co-physical activity is a vital element in fostering as well as maintaining positive physical activity habits in teenagers.

The study participants often reported that they maintained a healthy food environment at home to instil healthy dietary behaviours in adolescents. Several fathers discussed having dinner on a regular basis with adolescents. This is in line with a cross-sectional study conducted in the US where 47% of the Latino adolescents (*n* = 191; aged 10–14 years) claimed having family meals ≥ 7 times a week with their fathers [38]. A recent systematic review recognizes family mealtime as an important food-centred setting which allows both fathers and mothers to engage and interact with their children as well as facilitate the transfer of parental nutrition knowledge through various parental feeding practices [36]. One such feeding practice frequently employed by our study participants was implementation of food rules. Commonly implemented rules included not watching television while consuming meals and restricting the intake of junk food. In the same way, Khandpur and colleagues reported that 81.5% fathers (*n* = 40) had mealtime rules which were applicable to location of meals, use of electronic gadgets, dining etiquette, types of food and quantity of some foods, particularly snacks [82]. Fascinatingly, the use of restrictive snacking rules by Dutch fathers was significantly and negatively related to adolescents’ snack intake [83]. Even the recent US-based study reveals that adolescents’ intake of fast food and sugar sweetened beverages was lower when their fathers set lower limits for fast food and sugar sweetened beverage intake [38]. In addition to setting food rules, the interviewees also talked about limiting the availability of unhealthy food and drinks in the household, a food parenting practice also implemented by Latino fathers [79]. Contrary to these findings, American fathers reported that healthy food provisioning was not their responsibility and they mostly engaged in providing unhealthy foods like ultra-processed food to their teenagers [37].

Apart from providing a healthy family food environment, fathers also claimed to disseminate food and nutrition knowledge to their teenagers. They informed the teenagers about nutritious foods and its benefits as well as explained the repercussions associated with rampant consumption of energy-dense, nutrient-poor foods and sugar-sweetened beverages. Few fathers also reported that they engaged their children in meal preparation on weekends or holidays to impart essential food skills so that the adolescents can lead a healthy, independent life as adults. Echoing these views, fathers in two more qualitative studies conducted in the US also reported imparting both declarative and procedural nutrition knowledge to their children [79, 82].

Despite sharing nutrition knowledge, fathers faced a hard time in establishing healthy dietary behaviours in adolescents because of their penchant for unhealthy food and drinks. Parallel to these findings, Latino fathers expressed concern over their adolescents’ unhealthy dietary intake, particularly, the consumption of sugar sweetened beverages [79]. Indeed, urban Indian adolescents exhibit obesogenic dietary habits including low intake of fruits and vegetables and increasing consumption of fast food and carbonated beverages [3, 84]. In addition, Indian adolescents lead a sedentary lifestyle which was demonstrated by physical inactivity and too much screen time [85, 86]. This inactive lifestyle made our interviewees apprehensive as it could have detrimental effects on their adolescents’ physical and mental health. Correspondingly, Australian [87], British [88] and Mexican-American [79] fathers also expressed a high degree of concern and frustration regarding their teenagers’ physical inactivity and screentime.

The lack of time due to long work hours barred our study participants from taking adequate care of the adolescents on a regular basis, a barrier consistently reported in the literature [31, 63, 79, 89]. Additionally, mostly fathers from lower and middle socio-economic strata cited financial constraints as a major hindrance in providing nutritious food to adolescents. Reiterating these views, rural Pakistani fathers of children (age under five years) reported that poverty negatively impacted their parenting capabilities as lack and instability of money deterred them from purchasing food, clothes, and toys for their little ones on several occasions [77].

Our participants were well-informed about both forms of malnutrition (i.e. undernutrition as well as overnutrition). However, none of them were aware of any current government-led adolescent health programs. Sadly, overseas data also indicate fathers’ low levels of awareness regarding behavioural health programs for their children [90–92]. This ignorance of parents could be partially accountable for sluggish performances of several adolescent welfare programs in India [93, 94], thus accentuating the need for fatherhood education programs for Indian fathers. The interviewees exhibited interest in receiving more information regarding adolescents’ dietary and physical activity requirements; further reinforcing the necessity of paternal education programs.

Amidst the backdrop of limited evidence on paternal parenting practices in lower- and middle- income countries, this was the first research inquiry involving Indian fathers to identify their views and experiences of nurturing healthy dietary and activity behaviours in adolescents. Although this study provides rich, in-depth first-hand information on Indian fathers’ perspectives on parenting, it is not without limitations. First, the predominance of fathers from the higher socio-economic class within the sample could have minimized the generalizability of the findings. However, except for the financial barrier sub-theme, the remaining themes and sub-themes did not vary according to fathers’ socio-economic strata. Interestingly, Walsh and colleagues also did not observe any substantial differences based on paternal socio-economic position in Australian fathers’ perceptions about the diet and physical activity behaviours of their young children [64]. Nevertheless, the perspectives of fathers living in low-income settings are also vital for developing lifestyle interventions and therefore, prospective studies should also explore the views of Indian fathers belonging to economically weaker sections of the society on nurturing healthy behaviours in adolescents. Second, the emerging findings cannot be generalised to the Indian fathers on the whole because this investigation was based typically on urban fathers. Perhaps, to improve the generalizability, there is a need to replicate this inquiry in a rural setting. Third, the use of convenience and snowball sampling techniques could have also impacted the generalizability of our results. Nonetheless, qualitative researchers endorse the use of both these deliberate sampling techniques in qualitative investigations since the purpose of the qualitative investigations is not to generalize but rather to furnish rich, in-depth data on the topic of interest [95–97]. Fourth, this research inquiry only captured fathers’ views and it is quite possible that Indian mothers’ and adolescents’ views on paternal parenting may be different. Thus, to gain a comprehensive view on the current topic, future research should also investigate the perspectives of both Indian mothers and adolescents on Indian fathers’ parenting practices which could potentially inform effective and sustainable family-focussed nutrition and physical activity interventions.

## Conclusions

This qualitative inquiry addresses the dearth of research on paternal parenting practices to inform effective family-focussed lifestyle interventions and public health policies for the well-being of adolescents. On the whole, this inquiry reveals that Indian fathers played a crucial role in instilling healthy dietary and physical activity behaviour in their adolescents through various parenting practices such as purchasing nutritious food, enforcing food rules, managing the home food environment, disseminating nutrition-related knowledge, and encouraging adolescents to participate in moderate-to-vigorous intensity outdoor sports. Additionally, the fathers also shared their concern over adolescents’ unhealthy diet and lifestyle. The fathers also expressed their frustration on not spending enough time with their adolescents because of long working hours, underscoring the need to investigate existing workplace policies in India. Fathers’ ignorance about on-going government-led programs and policies for adolescents reiterates the need to involve fathers while designing family-focussed adolescent health interventions which could potentially ensure effective delivery of these interventions.

## Data Availability

The datasets used and/or analysed during the current study are available from the corresponding author on reasonable request.

## References

[1] Adolescent. nutrition [https://www.unicef.org/india/what-we-do/adolescent-nutrition] (2021). Accessed 21st March 2021.

[2] Peltzer K, Pengpid S (2012). Fruits and vegetables Consumption and Associated Factors among In-School adolescents in five southeast Asian countries. Int J Environ Res Public Health.

[3] Rathi N, Riddell L, Worsley A (2017). Food consumption patterns of adolescents aged 14–16 years in Kolkata, India. Nutr J.

[4] Rathi N, Riddell L, Worsley A (2018). Urban Indian adolescents practise unhealthy dietary behaviours. Br Food J.

[5] Katapally TR, Goenka S, Bhawra J, Mani S, Krishnaveni GV, Kehoe SH, Lamkang AS, Raj M, McNutt K. Results from India’s 2016 Report Card on Physical Activity for Children and Youth. J Phys Act Health. 2016;13(s2):176–S182. 10.1123/jpah.2016. 10.1123/jpah.2016-0393 27848756

[6] Kishore J (2020). Physical inactivity in adolescents: Manmade Epidemic. Indian J Youth Adolesc Health.

[7] Weihrauch-Blüher S, Wiegand S (2018). Risk factors and implications of childhood obesity. Curr Obes Rep.

[8] Sahoo K, Sahoo B, Choudhury AK, Sofi NY, Kumar R, Bhadoria AS (2015). Childhood obesity: causes and consequences. J Family Med Prim Care.

[9] Janssen I, Boyce WF, Simpson K, Pickett W (2006). Influence of individual- and area-level measures of socioeconomic status on obesity, unhealthy eating, and physical inactivity in Canadian adolescents. Am J Clin Nutr.

[10] National Family Health Survey (NFHS-5.) 2019–2021 - National Factsheet [http://rchiips.org/nfhs/factsheet_NFHS-5.shtml] (2022). Accessed 25 May 2023.

[11] Larson N, Story M (2009). A review of environmental influences on food choices. Ann Behav Med.

[12] Khandpur N, Charles J, Davison KK (2016). Fathers’ perspectives on coparenting in the context of child feeding. Child Obes.

[13] Rathi N, Riddell L, Worsley A (2016). What influences urban Indian secondary school students’ food consumption?– a qualitative study. Appetite.

[14] Gustafson SL, Rhodes RE (2006). Parental correlates of physical activity in children and early adolescents. Sport Med.

[15] Cheng LA, Mendonça G (2014). Farias Júnior JCd: physical activity in adolescents: analysis of the social influence of parents and friends. J De Pediatria.

[16] Trost SG, Loprinzi PD (2011). Parental influences on physical activity behavior in children and adolescents: a brief review. Am J Lifestyle Med.

[17] McPhie S, Skouteris H, Daniels L, Jansen E (2014). Maternal correlates of maternal child feeding practices: a systematic review. Matern Child Nutr.

[18] Johnson SL, Goodell LS, Williams K, Power TG, Hughes SO (2015). Getting my child to eat the right amount. Mothers’ considerations when deciding how much food to offer their child at a meal. Appetite.

[19] Fisk CM, Crozier SR, Inskip HM, Godfrey KM, Cooper C, Robinson SM (2011). Influences on the quality of young children’s diets: the importance of maternal food choices. Br J Nutr.

[20] Kakar S (1978). The Inner World: a Psycho-Analytic Study of Childhood and Society in India.

[21] Campbell KJ, Crawford DA, Salmon J, Carver A, Garnett SP, Baur LA (2007). Associations between the home food environment and obesity-promoting eating behaviors in adolescence. Obes.

[22] Beagan B, Chapman GE, D’Sylva A, Bassett BR (2008). `It’s just easier for me to do it’: rationalizing the Family Division of Foodwork. Sociol.

[23] Saraff A, Srivastava HC (2008). Envisioning fatherhood: Indian fathers’ perceptions of an ideal father. Popul Rev.

[24] Mbekenga CK, Lugina HI, Christensson K, Olsson P (2011). Postpartum experiences of first-time fathers in a Tanzanian suburb: a qualitative interview study. Midwifery.

[25] Sriyasak A, Almqvist A-L, Sridawruang C, Neamsakul W, Häggström-Nordin E (2016). The New Generation of Thai fathers: breadwinners involved in parenting. Am J Mens Health.

[26] Bianchi SM (2000). Maternal employment and time with children: dramatic change or surprising continuity?. Demogr.

[27] Gahlawat N, Phogat RS, Kundu SC (2019). Evidence for life satisfaction among Dual-Career couples: the interplay of job, Career, and family satisfaction in relation to Workplace Support. J Fam Issues.

[28] Sooryamoorthy R (2012). The Indian family: needs for a revisit. J Comp Fam Stud.

[29] Tagnan JN, Amponsah O, Takyi SA, Azunre GA, Braimah I (2022). A view of urban sprawl through the lens of family nuclearisation. Habitat Int.

[30] Ali S, George A (2022). Redressing urban isolation: a multi-city case study in India. J Urban Manag.

[31] Sriram R (2011). The role of fathers in children’s lives: a view from urban India. Child Educ.

[32] Awini S, Hoeschle-Zeledon I, Kizito F, Saaka M (2023). Fathers’ level of involvement in childcare activities and its association with the diet quality of children in Northern Ghana. Public Health Nutr.

[33] Schoppe-Sullivan SJ, Shafer K, Olofson EL, Kamp Dush CM (2021). Fathers’ parenting and coparenting behavior in dual-earner families: contributions of traditional masculinity, father nurturing role beliefs, and maternal gate closing. Psychol Men Masc.

[34] Novianti R, Suarman, Islami N (2023). Parenting in Cultural Perspective. J Ethn Cult Stud.

[35] Offer S, Kaplan D (2021). The New Father between ideals and practices: new masculinity ideology, gender role attitudes, and fathers’ involvement in Childcare. Soc Probl.

[36] Rahill S, Kennedy A, Kearney J (2020). A review of the influence of fathers on children’s eating behaviours and dietary intake. Appetite.

[37] Fielding-Singh P (2017). Dining with dad: fathers’ influences on family food practices. Appetite.

[38] Baltaci A, Alvarez de Davila S, Reyes Peralta AO, Laska MN, Larson N, Hurtado GA, Reicks M. Adolescent-reported latino fathers’ Food Parenting practices and Family Meal frequency are Associated with Better Adolescent Dietary Intake. Int J Environ Res Public Health. 2021;18(15). 10.3390/ijerph18158226. 10.3390/ijerph18158226 PMC8346089 34360517

[39] Litchford A, Savoie Roskos MR, Wengreen H (2020). Influence of fathers on the feeding practices and behaviors of children: a systematic review. Appetite.

[40] Morgan PJ, Young MD (2017). The influence of fathers on children’s physical activity and dietary behaviors: insights, recommendations and future directions. Curr Obes Rep.

[41] Lloyd AB, Lubans DR, Plotnikoff RC, Collins CE, Morgan PJ (2014). Maternal and paternal parenting practices and their influence on children’s adiposity, screen-time, diet and physical activity. Appetite.

[42] Bronte-Tinkew J, Moore KA, Carrano J (2006). The Father-Child Relationship, parenting styles, and adolescent risk behaviors in Intact families. J Fam Issues.

[43] Allport BS, Johnson S, Aqil A, Labrique AB, Nelson T, Kc A, Carabas Y, Marcell AV (2018). Promoting Father involvement for child and Family Health. Acad Pediatr.

[44] Panter-Brick C, Burgess A, Eggerman M, McAllister F, Pruett K, Leckman JF (2014). Practitioner review: engaging fathers–recommendations for a game change in parenting interventions based on a systematic review of the global evidence. J Child Psychol Psychiatry.

[45] Schoeppe S, Röbl M, Liersch S, Krauth C, Walter U (2016). Mothers and fathers both matter: the positive influence of parental physical activity modeling on children’s leisure-time physical activity. Pediatr Exerc Sci.

[46] Rainham DG, Bennett M, Blanchard CM, Kirk SFL, Rehman L, Stone M, Stevens D. Parents and children should be more active together to address physical inactivity and sedentary behaviours. Front Public Health. 2022; 10. 10.3389/fpubh.2022.633111 PMC9024119 35462818

[47] Ha AS, Ng JYY, Lonsdale C, Lubans DR, Ng FF (2019). Promoting physical activity in children through family-based intervention: protocol of the active 1 + FUN randomized controlled trial. BMC Public Health.

[48] Davison KK, Kitos N, Aftosmes-Tobio A, Ash T, Agaronov A, Sepulveda M, Haines J (2018). The forgotten parent: fathers’ representation in family interventions to prevent childhood obesity. Prev Med.

[49] Morgan PJ, Young MD, Lloyd AB, Wang ML, Eather N, Miller A, Murtagh EM, Barnes AT, Pagoto SL. Involvement of fathers in pediatric obesity treatment and prevention trials: a systematic review. Pediatr. 2017;139(2). 10.1542/peds.2016-2635. 10.1542/peds.2016-2635 PMC6200318 28130430

[50] Lloyd AB, Lubans DR, Plotnikoff RC, Morgan PJ (2015). Paternal lifestyle-related parenting practices mediate changes in children’s dietary and physical activity behaviors: findings from the healthy dads, healthy kids community randomized controlled trial. J Phys Act Health.

[51] Morgan PJ, Collins CE, Plotnikoff RC, Callister R, Burrows T, Fletcher R, Okely AD, Young MD, Miller A, Lloyd AB (2014). The ‘Healthy dads, healthy kids’ community randomized controlled trial: a community-based healthy lifestyle program for fathers and their children. Prev Med.

[52] O’Connor TM, Beltran A, Musaad S, Perez O, Flores A, Galdamez-Calderon E, Isbell T, Arredondo EM, Parra Cardona R, Cabrera N (2020). Feasibility of targeting hispanic fathers and children in an obesity intervention: Papás Saludables Niños Saludables. Child Obes.

[53] Morgan PJ, Grounds JA, Ashton LM, Collins CE, Barnes AT, Pollock ER, Kennedy S-L, Rayward AT, Saunders KL, Drew RJ (2022). Impact of the ‘Healthy youngsters, healthy dads’ program on physical activity and other health behaviours: a randomised controlled trial involving fathers and their preschool-aged children. BMC Public Health.

[54] Denzin NK, Lincoln YS. Introduction: The discipline and practice of qualitative research. In: *The Sage handbook of qualitative research* 4th edn. Edited by Denzin NK, Lincoln YS. Thousand Oaks, CA: Sage Publications Inc; 2011.

[55] Creswell JW, Poth CN (2013). Qualitative inquiry and research design: choosing among five approaches.

[56] Andrews T (2012). What is social constructionism?. Grounded Theory Rev.

[57] Tong A, Sainsbury P, Craig J (2007). Consolidated criteria for reporting qualitative research (COREQ): a 32-item checklist for interviews and focus groups. Int J Qual Health Care.

[58] Peasgood T, Bourke M, Devlin N, Rowen D, Yang Y, Dalziel K (2023). Randomised comparison of online interviews versus face-to-face interviews to value health states. Soc Sci Med.

[59] Shapka JD, Domene JF, Khan S, Yang LM (2016). Online versus in-person interviews with adolescents: an exploration of data equivalence. Comput Hum Behav.

[60] Cachia M, Millward L (2011). The telephone medium and semi-structured interviews: a complementary fit. Qual Res Organ Manag.

[61] Lechuga VM (2012). Exploring culture from a distance: the utility of telephone interviews in qualitative research. Int J Qual Stud Educ.

[62] Żadkowska M, Dowgiałło B, Gajewska M, Herzberg-Kurasz M, Kostecka M (2022). The sociological confessional: a reflexive process in the transformation from face-to-face to online interview. Int J Qual Methods.

[63] Bilal S, Spigt M, Czabanowska K, Mulugeta A, Blanco R, Dinant G (2016). Fathers’ perception, practice, and challenges in Young Child Care and Feeding in Ethiopia. Food Nutr Bull.

[64] Walsh AD, Hesketh KD, van der Pligt P, Cameron AJ, Crawford D, Campbell KJ (2017). Fathers’ perspectives on the diets and physical activity behaviours of their young children. PLoS ONE.

[65] Saunders B, Sim J, Kingstone T, Baker S, Waterfield J, Bartlam B, Burroughs H, Jinks C (2018). Saturation in qualitative research: exploring its conceptualization and operationalization. Qual Quant.

[66] Sandelowski M (2000). Whatever happened to qualitative description?. Res Nurs Health.

[67] King N. Using templates in the thematic analysis of text. In: *Essential guide to qualitative methods in organizational research* edn. Edited by Cassell C, Symon G. London, UK: Sage Publications Ltd.; 2004: 256–268.

[68] Marques JF, McCall C (2005). The application of interrater reliability as a solidification instrument in a phenomenological study. Qual Rep.

[69] Sahu M, Das A, Chattopadhyay B, Paul B, Bhattacharyya M (2021). Revised socioeconomic status scales for the year 2021: Updation based on latest base year series 2016. DY Patil J Health Sci.

[70] Bahadur A, Dhawan N (2008). Social value of parents and children in joint and nuclear families. J Indian Acad Appl Psychol.

[71] Lai-Yeung WLT (2011). Nutrition Education for adolescents: principals’ views. Asia Pac J Clin Nutr.

[72] Pendergast D, Garvis S, Kanasa H (2011). Insight from the Public on Home Economics and Formal Food Literacy. Fam Consum Sci Res J.

[73] Rathi N, Riddell L, Worsley A (2017). Food and nutrition education in private Indian secondary schools. Health Educ.

[74] Rathi N, Riddell L, Worsley A (2019). Parents’ and teachers’ critique of nutrition education in Indian secondary schools. Health Educ.

[75] Slater J (2013). Is cooking dead? The state of Home Economics Food and Nutrition education in a Canadian province. Int J Consum Stud.

[76] Snethen JA, Broome ME, Kelber S, Leicht S, Joachim J, Goretzke M (2008). Dietary and physical activity patterns: examining fathers’ perspectives. J Spec Pediatr Nurs.

[77] Jeong J, Siyal S, Fink G, McCoy DC, Yousafzai AK (2018). His mind will work better with both of us: a qualitative study on fathers’ roles and coparenting of young children in rural Pakistan. BMC Public Health.

[78] Kansiime N, Atwine D, Nuwamanya S, Bagenda F (2017). Effect of male involvement on the Nutritional Status of Children Less Than 5 years: A Cross Sectional Study in a Rural Southwestern District of Uganda. J Nutr Metab.

[79] Zhang Y, Hurtado GA, Flores R, Alba-Meraz A, Reicks M (2018). Latino fathers’ perspectives and parenting practices regarding eating, physical activity, and screen time behaviors of early adolescent children: Focus Group findings. J Acad Nutr Diet.

[80] Zahra J, Sebire SJ, Jago R (2015). He’s probably more Mr. sport than me– a qualitative exploration of mothers’ perceptions of fathers’ role in their children’s physical activity. BMC Pediatr.

[81] Isgor Z, Powell LM, Wang Y (2013). Multivariable analysis of the association between fathers’ and youths’ physical activity in the United States. BMC Public Health.

[82] Khandpur N, Charles J, Blaine RE, Blake C, Davison K (2016). Diversity in fathers’ food parenting practices: a qualitative exploration within a heterogeneous sample. Appetite.

[83] Gevers DWM, van Assema P, Sleddens EFC, de Vries NK, Kremers SPJ (2015). Associations between general parenting, restrictive snacking rules, and adolescent’s snack intake. The roles of fathers and mothers and interparental congruence. Appetite.

[84] Jain A, Mathur P (2020). Intake of Ultra-processed Foods among adolescents from low- and middle-income families in Delhi. Indian Pediatr.

[85] Nancy S, Rahman KM, Kumar SS, Sofia S, Robins MA (2022). Reasons and solutions for unhealthy food consumption and physical inactivity among school-going adolescents: a sequential mixed-methods study in Puducherry, South India. J Family Med Prim Care.

[86] Lyngdoh M, Akoijam BS, Agui RS, Sonarjit Singh K (2019). Diet, Physical Activity, and Screen Time among School students in Manipur. Indian J Community Med.

[87] Hattersley LA, Shrewsbury VA, King LA, Howlett SA, Hardy LL, Baur LA (2009). Adolescent-parent interactions and attitudes around screen time and sugary drink consumption: a qualitative study. Int J Behav Nutr Phys Act.

[88] McMichael L, Farič N, Newby K, Potts HWW, Hon A, Smith L, Steptoe A, Fisher A (2020). Parents of adolescents perspectives of physical activity, Gaming and virtual reality: qualitative study. JMIR Serious Games.

[89] Rakotomanana H, Walters CN, Komakech JJ, Hildebrand D, Gates GE, Thomas DG, Fawbush F, Stoecker BJ (2021). Fathers’ involvement in child care activities: qualitative findings from the highlands of Madagascar. PLoS ONE.

[90] Tully LA, Piotrowska PJ, Collins DAJ, Mairet KS, Black N, Kimonis ER, Hawes DJ, Moul C, Lenroot RK, Frick PJ (2017). Optimising child outcomes from parenting interventions: fathers’ experiences, preferences and barriers to participation. BMC Public Health.

[91] Lechowicz ME, Jiang Y, Tully LA, Burn MT, Collins DAJ, Hawes DJ, Lenroot RK, Anderson V, Doyle FL, Piotrowska PJ (2019). Enhancing Father Engagement in Parenting Programs: translating Research into Practice recommendations. Aust Psychol.

[92] Frank TJ, Keown LJ, Dittman CK, Sanders MR (2015). Using Father Preference Data to increase Father Engagement in evidence-based parenting programs. J Child Fam Stud.

[93] Sivagurunathan C, Umadevi R, Rama R, Gopalakrishnan S (2015). Adolescent health: present status and its related programmes in India. Are we in the right direction?. J Clin Diagn Res.

[94] Kasthuri A (2018). Challenges to Healthcare in India - The Five A’s. Indian J Community Med.

[95] Luborsky MR, Rubinstein RL (1995). Sampling in qualitative research: Rationale, Issues, and methods. Res Aging.

[96] Robinson OC (2014). Sampling in interview-based qualitative research: a theoretical and practical guide. Qual Res Psychol.

[97] Korstjens I, Moser A (2018). Series: practical guidance to qualitative research. Part 4: trustworthiness and publishing. Eur J Gen Pract.

